# Deep learning model for distinguishing Mayo endoscopic subscore 0 and 1 in patients with ulcerative colitis

**DOI:** 10.1038/s41598-023-38206-6

**Published:** 2023-07-13

**Authors:** Ji Eun Kim, Yoon Ho Choi, Yeong Chan Lee, Gyeol Seong, Joo Hye Song, Tae Jun Kim, Eun Ran Kim, Sung Noh Hong, Dong Kyung Chang, Young-Ho Kim, Soo-Yong Shin

**Affiliations:** 1 Department of Medicine, Samsung Medical Center, Sungkyunkwan University School of Medicine, 81 Irwon-Ro, Gangnam-gu, Seoul, 06351 South Korea; 2 Department of Artificial Intelligence and Informatics Research, Mayo Clinic, Jacksonville, FL USA; 3 Department of Digital Health, Samsung Advanced Institute for Health Sciences and Technology, Sungkyunkwan University, 81 Irwon-Ro, Gangnam-gu, Seoul, 06351 South Korea; 4 Research Institute for Future Medicine, Samsung Medical Center, Seoul, South Korea; 5 Department of Medicine, Nowon Eulji Medical Center, Eulji University, Seoul, South Korea

**Keywords:** Biotechnology, Gastroenterology

## Abstract

The aim of this study was to address the issue of differentiating between Mayo endoscopic subscore (MES) 0 and MES 1 using a deep learning model. A dataset of 492 ulcerative colitis (UC) patients who demonstrated MES improvement between January 2018 and December 2019 at Samsung Medical Center was utilized. Specifically, two representative images of the colon and rectum were selected from each patient, resulting in a total of 984 images for analysis. The deep learning model utilized in this study consisted of a convolutional neural network (CNN)-based encoder, with two auxiliary classifiers for the colon and rectum, as well as a final MES classifier that combined image features from both inputs. In the internal test, the model achieved an F1-score of 0.92, surpassing the performance of seven novice classifiers by an average margin of 0.11, and outperforming their consensus by 0.02. The area under the receiver operating characteristic curve (AUROC) was calculated to be 0.97 when considering MES 1 as positive, with an area under the precision-recall curve (AUPRC) of 0.98. In the external test using the Hyperkvasir dataset, the model achieved an F1-score of 0.89, AUROC of 0.86, and AUPRC of 0.97. The results demonstrate that the proposed CNN-based model, which integrates image features from both the colon and rectum, exhibits superior performance in accurately discriminating between MES 0 and MES 1 in patients with UC.

## Introduction

The treatment goal of ulcerative colitis (UC) is gradually becoming stricter after introduction of diverse biological agents. The current therapeutic golas for UC includes clinical remission and endoscopic remission^1,2^. Selecting Therapeutic Targets in Inflammatory Bowel Disease (STRIDE) I in 2015 defined endoscopic remission as a Mayo endoscopic subscore (MES) of 0 or 1^3^. However, STRIDE II in 2020 defined endoscopic remission as MES 0; therefore, patients with MES 1 need to step up to achieve MES 0^4–6^.

However, the step-up strategy cannot always result in MES 0, and it is not easy for MES 1 patients to step up which can lead to running short of available agents and having financial problems. And the most important issue is inaccuracy in the evaluation of MES. Distinguishing between MES 0, 1 versus MES 2 in UC patients is relatively straightforward, but discriminating between MES 0 and 1 poses a unique challenge as it requires careful discernment of subtle differences in endoscopic features. The severity of inter-/intra-observer variation among endoscopists in discriminating between MES 0 and 1 has been well-documented by previous studies^7–9^.

Recent breakthroughs in artificial intelligence (AI) have shown great potential in addressing the challenges of inter-/intra-observer variations and providing valuable support in the evaluation of endoscopic remission in real-world clinical practice. By leveraging AI techniques, studies focusing on endoscopic findings have emerged with the aim of overcoming the limitations of MES evaluation, as evidenced by recent publications^10–15^. These advancements hold promise for improving the accuracy and objectivity of endoscopic assessment, thereby enhancing the reliability and reproducibility of clinical outcomes.

Despite efforts in several institutions to enhance the objectivity of endoscopic evaluation through diverse study designs, there are no studies that have specifically focused on differentiating between MES 0 and MES 1, despite their clinical significance in evaluating endoscopic remission in patients with UC.

In this study, we built a convolutional neural network (CNN) based on the endoscopic features of UC patients with endoscopic improvement defined by an MES of ≤ 1. We focused on the different characteristics of MES 0 and MES 1 and developed an automated reading model for distinguishing between MES 0 and MES 1.

## Methods

### Patients

This single-center retrospective cohort study was conducted at Samsung Medical Center, a tertiary academic institution in Seoul, South Korea. The data of our cohort and study population were previously introduced to confirm the outcome of histologic remission^7^. All patients with UC at this center routinely visit the outpatient clinic and undergo routine colonoscopy. Our research institute has created an MES scoring protocol since 2018, including performing a two-point biopsy at work for all UC patients, and established an UC cohort of MES 0 or 1. Among the 1161 UC patients who underwent colonoscopy between January 2018 and December 2019 at this center, 492 patients with MES improvement (MES 0 or MES 1) were included and analyzed. The study protocol was reviewed and approved by the Samsung Medical Center (SMC) Institutional Review Board (IRB No. 2021-10-138-001), and conducted in accordance with the principles of the Declaration of Helsinki. We used only de-identified data routinely collected during hospital visits, so the requirement for informed consent was waived according to the rules of SMC IRB.

### Data collection

Two representative images of the colon and rectum, which appear to be the most severe, respectively were selected from the endoscopic images of 492 patients, and 984 images were obtained. Here, the data were split through random sampling into a training dataset of 452 persons/904 images for hyperparameter optimization and model construction and a test set of 40 persons/80 images for comparative experiments with the novice group. Supplementary Table 1 lists the composition of the entire dataset.

### Endoscopic evaluation

The endoscopic images were reviewed separately by three endoscopic specialists and scored from 0 to 3 according to the Mayo endoscopic subscore. MES is a component of the Mayo score, classifying mucosal inflammation based on a 4-point scale from 0 to 3 according to endoscopic findings (0: normal; 1: erythema, decreased vascular pattern, and mild friability; 2: marked erythema, absent vascular pattern, friability, and erosions; 3: ulceration and spontaneous bleeding)^8^. In cases of disagreement, the scores were recorded according to the consensus of two out of three reviewers. Endoscopic improvement was defined as an MES of 0 or 1, and complete endoscopic remission was defined as an MES of 0. The evaluation of the MES score was conducted using a colonoscope, specifically the Olympus CF-H260 or CF-Q260 model from Tokyo, Japan.

### Preprocessing

Our proposed method includes a preprocessing step to remove redundant information from the endoscopic image and ensuring that all images have the same size while preserving their aspect ratio (Supplementary Fig. 1). This preprocessing method involves several stages. First, the endoscopic image is converted to grayscale and then binarized using an arbitrary threshold. In our study, a threshold value of 25 was chosen to distinguish between redundant background and low foreground illumination. Next, we employ 8-connectivity connected component analysis to remove object groups other than the largest one from the binarized image. Subsequently, we crop the image by identifying the bounding box that tightly fits the largest object area. The cropped areas have varying sizes and aspect ratios (Supplementary Fig. 2). Therefore, to preserve the aspect ratio of the cropped image while transforming it into a square shape, we applied zero-padding to extend the shorter axis to match the length of the longest axis of the cropped area. Finally, the padded image is resized to a fixed size of 256 $\times$ 256 pixels. This preprocessing method provides a more refined foreground compared to fixed-length cropping or the vertical/horizontal projected histogram thresholding method^10^.

### Model architecture

**Figure 1:**
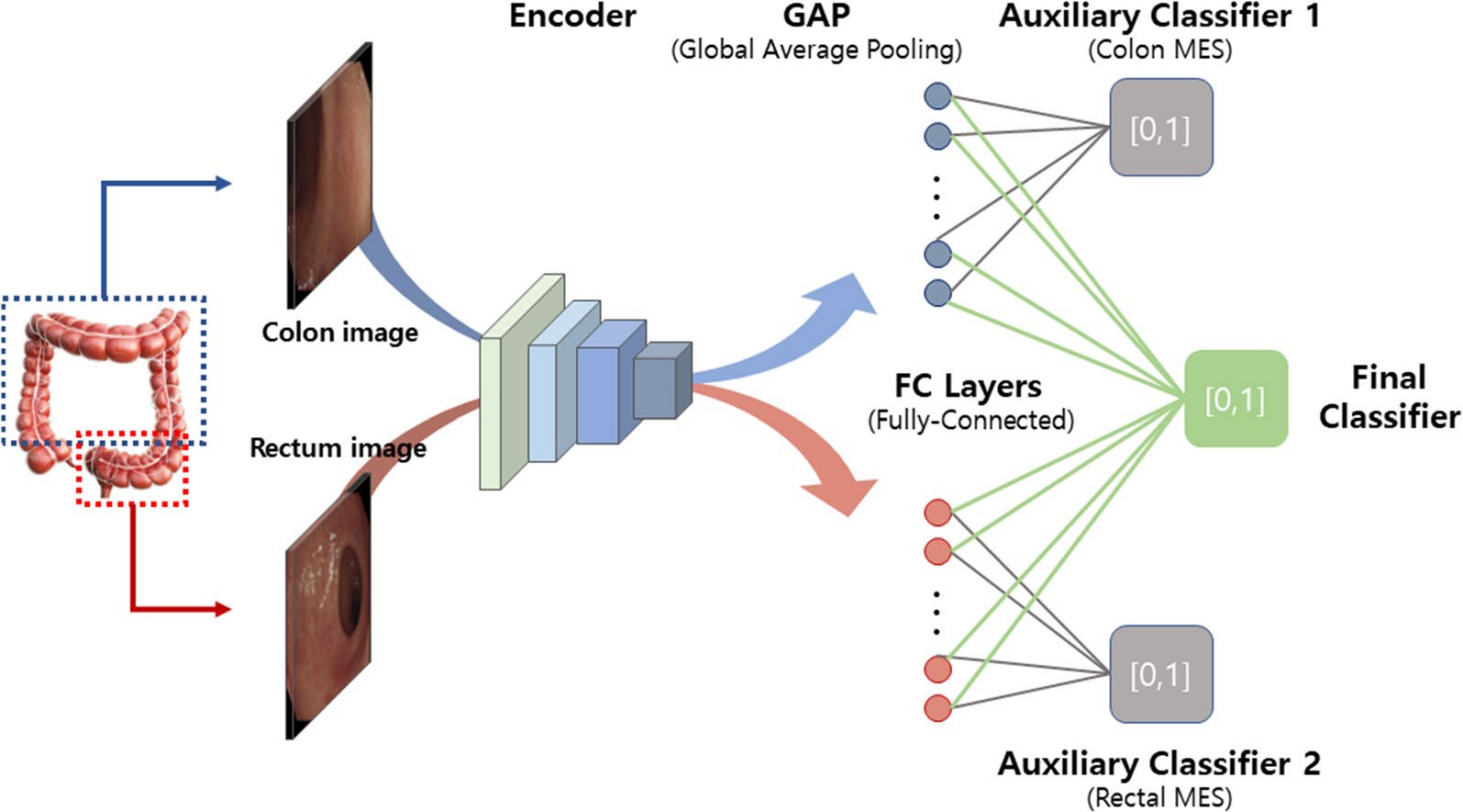
Artificial intelligence (AI) model architecture.

Our model comprises an encoder, two auxiliary classifiers, and a final classifier. Each auxiliary classifier outputs the MES score for each input image of the colon and rectum, and the final classifier integrates the two input image feature maps to predict the final MES score for the patient (Fig. 1 However, none of the previous deep learning studies have shown capability to distinguish MES 0 and MES 1.). The encoder extracts the features of the input image and works as a common backbone for the following three classifiers: The encoder consists of convolutional filters of CNN-based classification models pre-trained with the ImageNet database. Among various CNN structures, we chose VGG-16, which showed the best experimental performance^11,12^. The encoder was fine-tuned with our endoscopic data, and the training details and performance comparison are described in the “Settings” and “Experiments” sections, respectively. We added two auxiliary classifiers, independently predicting MES in the colon and rectum, to better guide the encoder over common features in the input endoscopic images. It consists of a binary classifier followed by global average pooling and a dense layer for encoded image features. This auxiliary classifier also enables MES classification even in limited conditions where only colon or rectum images are allowed. The final classifier predicts the patient's MES by aggregating the image feature maps of the colon and rectum extracted from the encoder. The architecture followed by the global average pooling and dense layer is the same as that of the auxiliary classifier but with twice the trainable parameters. The model is trained as a weighted sum of the binary cross-entropy losses calculated from each output of all classifiers, as shown in the following equation:

1 $$BCE\left(y, \widehat{y}\right) = -(ylog\left(\widehat{y}\right) +(1-y)\mathrm{log}(1-\widehat{y}))$$

2 $$Total \, loss = {BCE}_{final\, classifier} +\lambda ({BCE}_{auxiliary\, classifier\, for\, colon}+{BCE}_{auxiliary\, classifier\, for\, rectum})$$

In Eq. 2, $\lambda$ is the balancing factor of the loss functions between the final classifier and the two auxiliary classifiers. The additional backpropagation of the loss for each of the colon and rectal images to the shared encoder of the model allows it to learn a better representation for predicting the MES in the endoscopic image. For the experiment, we maintained the value $\lambda$ at 1.

### Settings

Data augmentation techniques of horizontal flip, vertical flip, rotation, zoom-in, and brightness adjustment were randomly applied for model training. For rich augmentation, these techniques were independently applied to each of the two input colons and rectal images of the model. The model was trained for 500 epochs with a batch size of 8 and optimized using Adam with an initial learning rate of 1e-5. All images input to the model in training and testing were standardized sample-wise. Our model was programmed in Python version 3.9.6 and TensorFlow version 2.8.0 with CUDA version 11.0. The model was trained and tested on a system with an NVIDIA GeForce RTX 2080 8 Gb GPU, 64 Gb RAM memory, and an Intel(R) Core(TM) i9-10850 K CPU @ 3.60 GHz CPU environment.

### Experiments

We conducted the following three experiments to evaluate and test our model.

(1) Twelve-fold cross-validation for representative model selection

The final hyperparameters and backbones were determined through 12-fold cross-validation using a training dataset of 452 patients/904 images from our improvement cohort. We evaluated the performance of the model using five different backbones: VGG16, MobileNet V2^13^, DenseNet121, EfficientNet B0, and ResNet50^13,16–18^, and conducted a performance comparison for the backbone according to initial weight settings, such as scratch learning and transfer learning. We also conducted a performance comparison experiment for optimal model architecture and hyperparameter exploration according to the loss weight λ in the range of 0 to 1.

(2) Internal test and performance comparison with the novice group

We compared the developed model with a novice group on 40 patients/80 images of the test dataset. The novice group consisted of 7 fellow doctors from the Department of Gastroenterology at our center. Their experience varied from three to seven months, with an average of five months. Each novice independently investigated each image pair of the colon and rectum in the test set according to the MES scoring guidelines, and the final MES was predicted. The consensus of MES prediction results for each novice was also calculated and compared with the AI model results.

(3) External test

To investigate the generalization capabilities of the model, we additionally conducted an external test using the Hyperkvasir dataset, a publicly available collection of endoscopic video and image^19^. The dataset includes colonoscopy images of MES grades 1,2, and 3, as well as images that are confounded between adjacent grades (0–1, 1–2, and 2–3). However, specific images representing the MES 0 are not included in the dataset. To address the absence of MES 0 data, we utilized videos from the Hyperkvasir dataset that were graded with Boston Bowel Preparation Scale (BBPS) score of 3. These videos represent intestines with perfectly clean mucosal conditions, free from residual stool or opaque liquid. From each of the eight BBPS 3 videos, we randomly sampled five still frames, excluding the initial and final 2-s intervals that commonly exhibited severe motion artifacts. This resulted in a total of 40 still images, which served as substitutes for MES 0 score images in the external test. The composition of the Hyperkvasir data used for external test can be found in Supplementary Table 5.

In this test, we focused on two important aspects of our model’s performance. Firstly, we assessed the classification performance of MES 0 and 1. Secondly, we evaluated the detection performance for all UC positive cases with MES scores higher than 1 (1–2, 2, 2–3, 3). The objective of the second experiment was to investigate whether our model possesses the capability to detect UC irrespective of its severity. Since the Hyperkvasir data in this experiment only included colon images, we sub-modeled and evaluated the auxiliary classifier specifically for colon images in our model.

### Evaluation metrics

To evaluate the classification results of our model, we applied standard classification metrics, such as accuracy, true positive ratio, sensitivity, the area under the receiver operating characteristic curve** (**AUROC), area under the precision-recall curve (AUPRC), and F1-score (Supplementary Table 2). Since the composition of the experimental data in this study shows class imbalance, we mainly compared the F1-score among them.

### Statistical analysis

Values are expressed as median (interquartile range) for continuous variables and number (%) for categorical variables. The chi-square, Fisher's exact, and Mann–Whitney U tests were used to compare the variables between the two groups. All statistical analyses were performed using the SPSS Statistics ver. 27.0 (IBM Corp, New York, NY, USA). Statistical significance was set at *p* < 0.05.

## Results

### Baseline characteristics

The baseline characteristics of UC patients with endoscopic improvement defined by an MES of 0 or 1 are summarized in Table 1. The median age was 48 (37–58) years, and 254 patients (51.6%) were male. The median duration of the disease was 549.0 (369.25–744.75) days; 253 patients (51.4%) showed complete endoscopic remission defined by a rectal MES of 0, and 220 patients (44.7%) showed complete endoscopic remission defined by a colon MES of 0. When evaluating the total MES score combined with colon and rectal MES, 217 patients (44.1%) achieved complete endoscopic remission.

**Table 1:** Baseline characteristics.

Total patients	All patients (492)
Age	48 (37–58)
Male, *n* (%)	254 (51.6)
Age at diagnosis	39.0 (29.0–49.0)
Disease duration (month)	78.0 (30.0–144.0)
Disease extent	
Proctitis	212 (43.1)
Left sided colitis	133 (27.0)
Pancolitis	147 (29.9)
BMI	22.96 (20.93–25.02)
Steroid use history	194 (39.4)
Current medication	
5-ASA, topical	99 (20.1)
5-ASA, oral	193 (39.2)
5-ASA, both	160 (32.5)
Steroid	6 (1.2)
Azathioprine	28 (5.7)
Immunomodulator	37 (7.5)
Laboratory finding	
WBC (/µL)	6060.0 (5140.0–7247.5)
Hemoglobin (g/dL)	14.0 (13.0–15.1)
Platelet count (×10^3^ µL)	247.0 (213.5–290.0)
ESR (mm/hr)	11.0 (5.0–20.0)
Albumin (g/dL)	4.6 (4.4–4.8)
CRP (mg/dL)	0.05 (0.03–0.11)
Endoscopic finding	
Colon MES 0	220 (44.7)
Rectal MES 0	253 (51.4)
Total MES 0	217 (44.1)
Follow up duration (day)	549.0 (369.25–744.75)

BMI, body mass index; 5-ASA, 5-aminosalicylic acid; ESR, Erythrocyte sedimentation rate; CRP, C-reactive protein; MES, Mayo endoscopic subscore.

### Representative model selected by 12-fold cross-validation

In the 12-fold cross-validation of the training dataset, VGG16 showed the best performance among the five backbones, with an average F1-score of 0.8738, accuracy of 0.8472, AUROC of 0.8699, and AUPRC of 0.8830 (Supplementary Table 3). Incorporating auxiliary classifiers into the model led to an average accuracy improvement of over 3% in all five backbones, and the optimal auxiliary loss weight λ was 1 (Supplementary Table 4). Supplementary Fig. 3 shows AUROC and AUPRC plots for the entire fold.

### Outcomes of internal test and comparison of our models with the novice group

**Figure 2:**
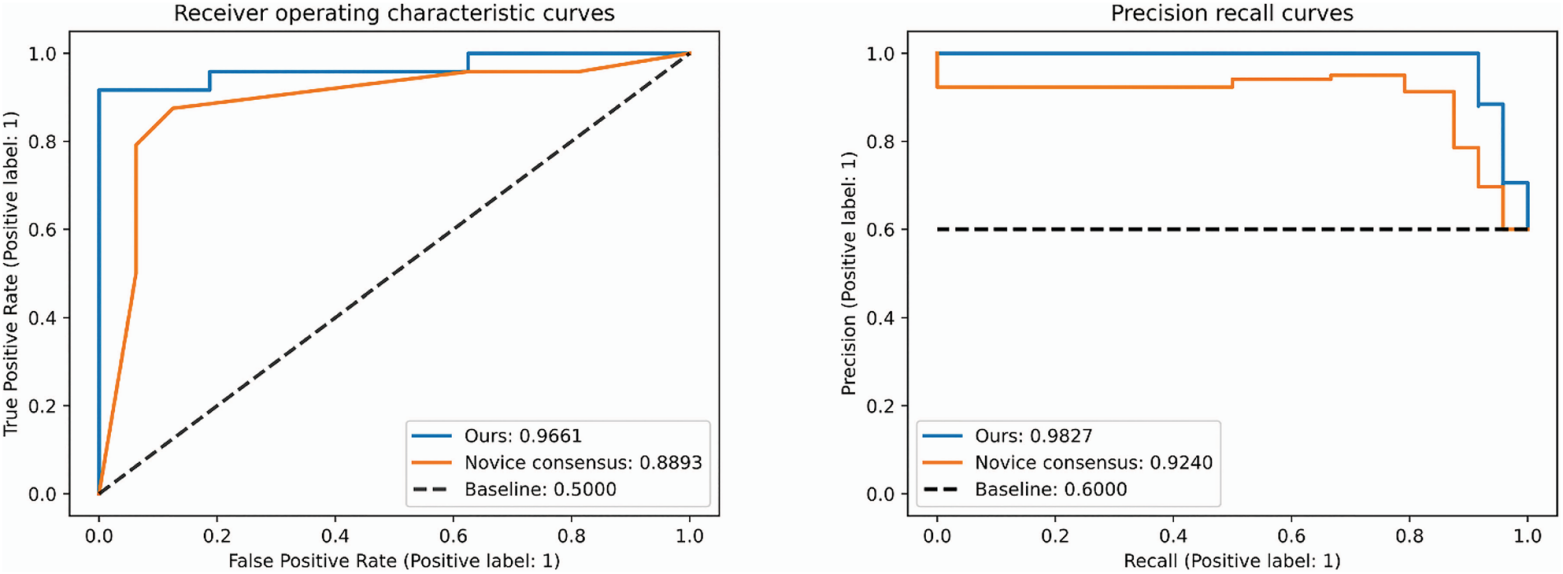
Area under the Receiver Operating Characteristic curve (AUROC) and Area under the Precision-Recall Curve (AUPRC) of our model and novice group on the internal test set.

**Figure 3:**
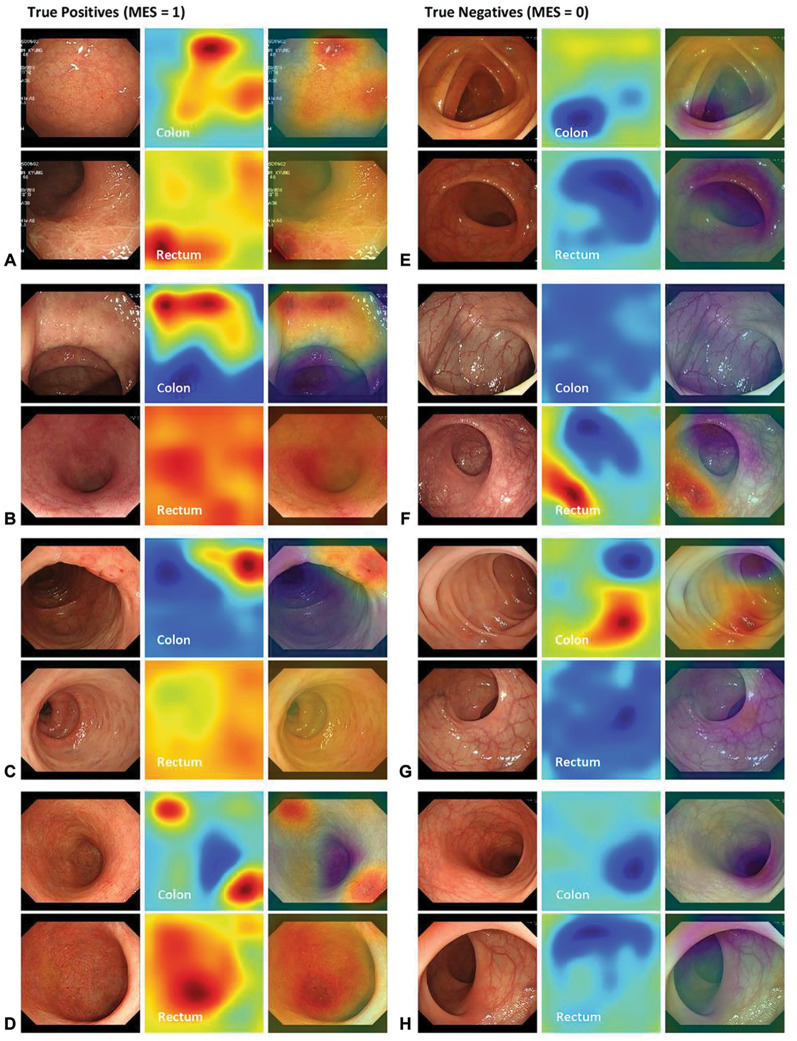
Examples of test images and their corresponding normalized class activation maps True positives (MES = 1) (**A**),(**B**),(**C**),(**D**). True negatives (MES = 0] (**E**),(**F**),(**G**), and (**H**) (https://pypi.org/project/matplotlib/3.5.3/).

The consensus of MES prediction results for each novice was also calculated and compared with our model. The AUROC and AUPRC of our model for the test set were 0.9661 and 0.9827, respectively, outperforming the consensus of the novices (Fig. 2). The consensus of the novice individuals and the novice group on the test set and the prediction results of our model are shown in Table 2 and are expressed as a confusion matrix (Supplementary Fig. 1). The average F1-score of the novice group was 80.26%, and their consensus showed an improved accuracy of 89.4%. In comparison, the test set F1-score of our model was 91.7%, showing the highest performance. Novices tended to overestimate disease exacerbations in terms of activity. Figure 3 shows the result of overlapping the normalized class activation map obtained for MES 1 on the two input images of the colon and rectum. MES 1 is shown in red, and MES 0 is shown in blue.

**Table 2:** Performance comparison between novices and our model in Internal test dataset.

	Accuracy	F1-Score	True positive ratio	Sensitivity
Novice 1	0.8250	0.8511	0.8696	0.8333
Novice 2	0.7250	0.7925	0.7241	0.8750
Novice 3	0.8500	0.8800	0.8462	0.9167
Novice 4	0.8000	0.8333	0.8333	0.8333
Novice 5	0.7500	0.7619	0.8889	0.6667
Novice 6	0.7000	0.7857	0.6875	0.9167
Novice 7	0.7000	0.7143	0.8333	0.6250
Consensus	0.8750	0.8936	0.9130	0.8750
Ours	0.9000	0.9167	0.9167	0.9167

### Outcomes of external test

**Figure 4:**
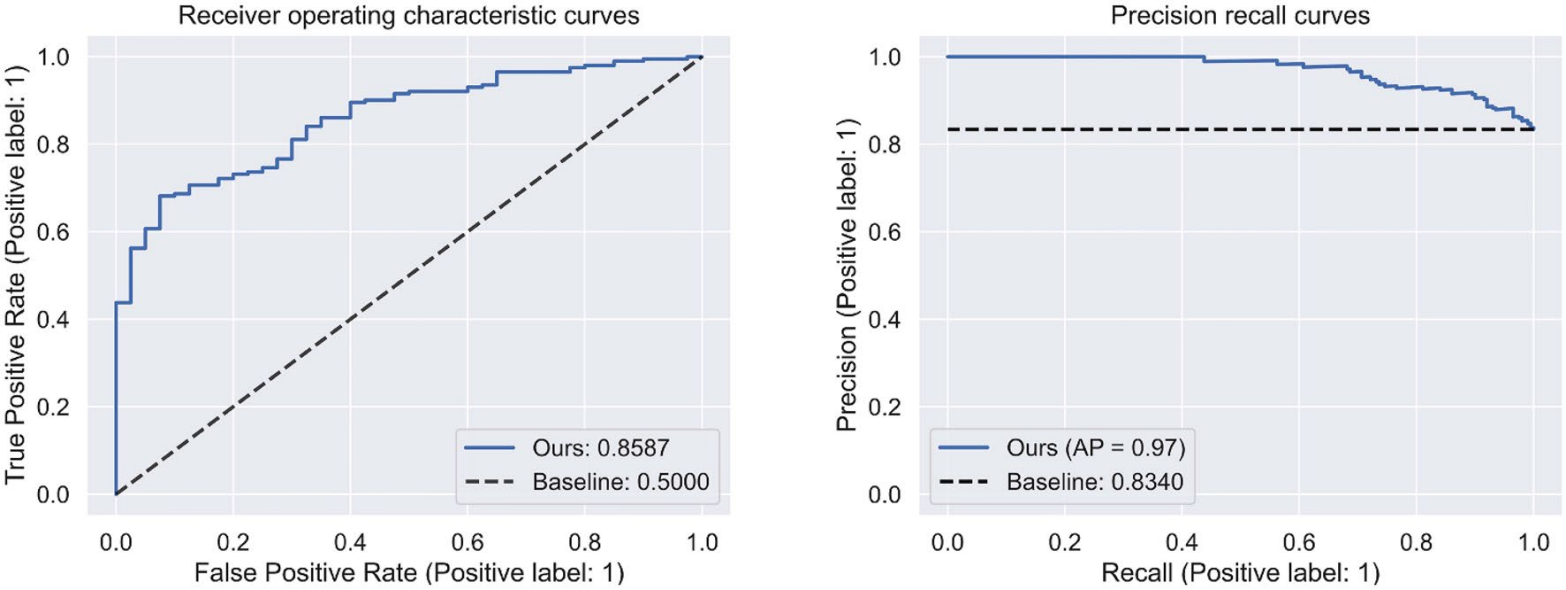
External test of predictive performance of our model.

Supplementary Table 5 shows the composition of the Hyperkvasir data used for external validation. We conducted an investigation into the classification performance of our model for Hyperkvasir's MES 0 and 1 data. Additionally, we examined the model's detection performance for UC, regardless of its severity, using only positive images with MES scores higher than 1. During the test for MES 0 and 1 classification, our auxiliary classifier demonstrated high performance, achieving an AUROC of 0.8587 and an AUPRC of 0.9696 (Fig. 4). Supplementary Fig. 5A showcases the model's excellent generalized performance, as depicted by the confusion matrix derived from Hyperkvasir's MES 0 and 1 data.

In the supplementary evaluation aiming to assess the model's ability to detect UC, our model classified only 35 out of 615 positive cases with MES scores higher than 1 as MES 0, as illustrated in Supplementary Fig. 5B.

## Discussion

Recognizing the necessity and difficulty of objectification in MES classification, many studies have been published to solve it through AI. In one study that examined whether MES was evaluated through a CNN, AUROC was 0.84 for MES ≥ 1, 0.85 for MES ≥ 2, and 0.85 for MES ≥ 3^20^. In another study, the developed deep learning model classified MES 0–1 and 2–3 with 94.5% accuracy, 89.2% sensitivity, and 96.3% specificity^21^. Most previous studies have focused on scoring and predicting MES 1 or higher or merging MES 0 and 1 into the same class^20,22,23^. Therefore, according to STRIDE II, they are not suitable for monitoring endoscopic remission. In addition, the performance of most existing studies was measured only with AUROC and accuracy, which have a high risk of giving distorted values for class-imbalanced data^24^. Our study conducted a more reliable performance analysis by including the AUPRC and the F1 score, which is the harmonic mean of precision and recall. Our end-to-end classification model, which accepts colon and rectum images simultaneously, is cost-effective and has performance advantages compared to creating two models that classify the images separately. In the VGG16-based classification model for colon (auxiliary classifier 1) and rectum images (auxiliary classifier 2), the average F1 scores were 0.6381 and 0.7296, respectively.

In addition, the structural strengths of our model can be summarized as follows. First, all classifiers are easy to implement with a simple structure consisting of global average pooling and one fully connected layer, requiring little additional cost from the backbone. Second, because the model capacity and performance are highly dependent on the backbone model, it is easy to upgrade performance by introducing an improved backbone. Third, the independent classification of colon or rectum images is possible through the sub-modeling of auxiliary classifiers.

Our study had some limitations. As it was a single-center retrospective cohort study, the number of patients was small; hence, the amount of data was inevitably small. To achieve the results of a multicenter study on deep learning to supplement this, an improvement cohort should be established in the same way as in our center. In clinical practice, it is important to perform MES scoring after observing the entire colon; however, it is technically difficult to make AI models by making these moving images rather than still images because of the data capacity. Recently, studies on clinical relapse through histological remission of UC patients have been actively published^25,26^. Furthermore, the findings of real-time video-based research have shown promising results in reducing the need for unnecessary biopsies and enhancing the accuracy of evaluations by integrating endoscopic and histological assessments^14^. However, reproducibility is difficult even if video data are technically constructed. The feasibility of practical implementation of this approach is deemed to require a considerable amount of time. Therefore, for applicability in actual clinical practice, as shown in our study, the appropriate method is to analyze the two images as a backbone and further analyze them using an auxiliary classifier, taking into account the properties of any image, and to give the final results.

We used only the MES score instead of Ulcerative Colitis Endoscopic Index of Severity (UCEIS) as an endoscopic evaluation tool. The simplification and clarity of deep learning algorithms can mitigate the occurrence of false positives and false negative. We thought that assessment of MES through grading is straightforward for deep learning, while UCEIS, due to the need for assigning points to each descriptor based on its definition, is complex and prone to errors. This study focused solely on evaluating the MES index, as it pertains to the changes brought about by STRIDE II, in order to draw conclusive research findings regarding MES0. Our future plans involve refining technology and developing algorithms that are free from UCEIS errors and are more sophisticated in nature.

Deep learning model is a valuable tool because of its low variation in results and reproducibility. Utilizing deep learning as a support system for clinical decision-making reduces the difference between observers; it is expected to be helpful for replacing "automated reading" now that MES 0 is suggested as a therapeutic goal in STIRIDE II. Discriminating between MES 0 and MES 1 is difficult for endoscopist in clinical practice. However, it is clear that patients with MES 0 have fewer clinical relapse, therefore it is necessary to distinguishing between the two because of saving drugs through accurate discrimination, and ultimately to decide on the patient’s treatmen plan. Through our model, this gap can be narrowed which can contribute to treatment improvement.

In conclusion, our study demonstrated the successful construction of a CNN utilizing endoscopic features of UC patients, specifically focusing on distinguishing between MES 0 and MES 1 for endoscopic improvement. Through rigorous development and testing, our automated reading model has proven its superiority over novice groups in an internal test and showcased excellent performance by external validation.

## Supplementary Information

Supplementary Information.

## Data Availability

The data underlying this article cannot be shared publicly given the privacy of the individuals who participated in the study. The data will be shared on reasonable request to the corresponding author.
