# Supplementary material for: Deep learning model for distinguishing Mayo endoscopic subscore 0 and 1 in patients with ulcerative colitis
Source: Sci Rep. 2023 Jul 13;13:11351. doi: 10.1038/s41598-023-38206-6 (PMC10344868; doi:10.1038/s41598-023-38206-6)

Deep learning model for distinguishing Mayo endoscopic subscore 0 and 1 in patients with ulcerative colitis

Ji Eun Kim[1,a] · Yoon Ho Choi[2,3,a] · Yeong Chan Lee[4] · Gyeol Seong[5] · Joo Hye Song[1] · Tae Jun Kim[1] · Eun Ran Kim[1] · Sung Noh Hong[1] · Dong Kyung Chang[1] · Young-Ho Kim[1,b] · Soo-Yong Shin[3,b]

Co-Correspondence

Young-Ho Kim, MD, PhD

Department of Medicine, Samsung Medical Center, Sungkyunkwan University School of Medicine, 81 Irwon-ro, Gangnam-gu, Seoul 06351, Korea

Phone: +82-2-3410-3409; Fax: +82-2-3410-6983; E-mail: bowelkim@gmail

&

Soo-Yong Shin, PhD

Department of Digital Health, Samsung Advanced Institute for Health Sciences & Technology, Sungkyunkwan University, 81 Irwon-ro, Gangnam-gu- 06351, Seoul, South Korea

Tel: +822-3410-1449; Fax: +822-3412-3994; Email: sooyong.shin@gmail.com

Supplementary Table 1. Internal data configuration

	Training		Test	
	**MES 0**	**MES 1**	**MES 0**	**MES 1**
**Number of patients (492)**	239	213	16	24
**Number of images (984)**	478	426	32	48

Supplementary Table 2. Definitions of evaluation metrics

Evaluation metric	Definition
**Accuracy**	$\frac{TP + TN}{TP + TN + FP + FN}$
**True positive ratio (TPR)**	$\frac{TP}{TP + FP}$
**Sensitivity**	$\frac{TP}{TP + FN}$
**AUROC**	$\int_0^1 Sensitivity(FPR)\mathrm{d}(FPR)$
**AUPRC**	$\int_0^1 TPR(Sensitivity)\mathrm{d}(Sensitivity)$
**F1-score**	$\frac{2TP}{2TP + FP + FN}$

TP, True positive; TN, True Negative; FP, False positive; FN, False Negative, FPR (False positive ratio) FP/(FP+TN)

Supplementary Table 3. 12-fold cross validation results for each backbone of the final model with auxiliary output (Auxiliary loss weight $\boldsymbol{\lambda}$ = 1.0)

**Backbone (# of parameters)**	**Accuracy**	**F1-score**	**True positive ratio**	**Sensitivity**	**AUROC**	**AUPRC**
**VGG16 (14,714,688)**	**0.8472±0.0318**	**0.8738±0.0241**	**0.8207±0.0501**	**0.9407±0.0588**	**0.8699±0.0442**	**0.8830±0.0459**
**ResNet50 (23,587,712)**	0.7455±0.0684	0.6813±0.0975	0.7491±0.0617	0.8308±0.0959	0.7351±0.0893	0.7565±0.1070
**DenseNet121 (7,037,504)**	0.6901±0.0620	0.7581±0.0450	0.6870±0.0740	0.8663±0.1096	0.6297±0.0984	0.6686±0.0898
**MobileNet V2 (2,257,984)**	0.6328±0.0309	0.7350±0.0303	0.6213±0.0334	0.9143±0.1060	0.5159±0.1022	0.5840±0.0778
**EfficientNet B0 (4,049,571)**	0.6906±0.0415	0.7528±0.0416	0.6929±0.0641	0.8533±0.1410	0.6326±0.0857	0.6680±0.0813

Supplementary Table 4. 12-fold cross-validation result of the final model architecture according to each Auxiliary loss weight (Backbone: VGG16)

**Auxiliary loss weight (λ)**	**Accuracy**	**F1-score**	**True positive ratio**	**Sensitivity**	**AUROC**	**AUPRC**
**0.1**	0.8038±0.0388	0.8658±0.0265	0.8018±0.0518	**0.9450±0.0377**	0.8616±0.0511	0.8766±0.0563
**0.2**	0.8316±0.0422	0.8564±0.0330	**0.8308±0.0653**	0.8937±0.0765	**0.8733±0.0394**	**0.8928±0.0500**
**0.3**	0.8250±0.0407	0.8524±0.0293	0.8205±0.0643	0.8938±0.0514	0.8474±0.0423	0.8703±0.0496
**0.4**	0.8384±0.0403	0.8654±0.0338	0.8166±0.0431	0.9253±0.0653	0.8709±0.0439	0.8870±0.0353
**0.5**	0.8362±0.0309	0.8613±0.0256	0.8272±0.0539	0.9060±0.0682	0.8693±0.0369	0.8866±0.0427
**0.6**	0.8272±0.0308	0.8590±0.0234	0.7993±0.0544	0.9369±0.0653	0.8683±0.0433	0.8920±0.0477
**0.7**	0.8408±0.0399	0.8673±0.0366	0.8153±0.0356	0.9289±0.0631	0.8713±0.0459	0.8803±0.0466
**0.8**	0.8317±0.0430	0.8618±0.0346	0.8034±0.0442	0.9333±0.0584	0.8615±0.0463	0.8812±0.0450
**0.9**	0.8406±0.0423	0.8671±0.0342	0.8231±0.0586	0.9210±0.0528	0.8706±0.0442	0.8768±0.0537
**1.0**	**0.8472±0.0318**	**0.8738±0.0241**	0.8207±0.0501	0.9407±0.0588	0.8699±0.0442	0.8830±0.0459

Supplementary Table 5. Configuration of external validation dataset (Hyperkvasir)

Dataset	Category	Amount (Images)
**Ulcerative colitis**	**Mayo Endoscopic Score (MES) ≥ 1**	**816**
	MES 1	201
	MES 1~2	11
	MES 2	443
	MES 2~3	28
	MES 3	133
**Quality of mucosal views**	**Boston Bowel Preparation Scale 2~3 (Regarded as MES 0)**	**40**

Supplementary Fig. 1. Overview of the preprocessing algorithm.

(URL: https://pypi.org/project/matplotlib/3.5.3/)

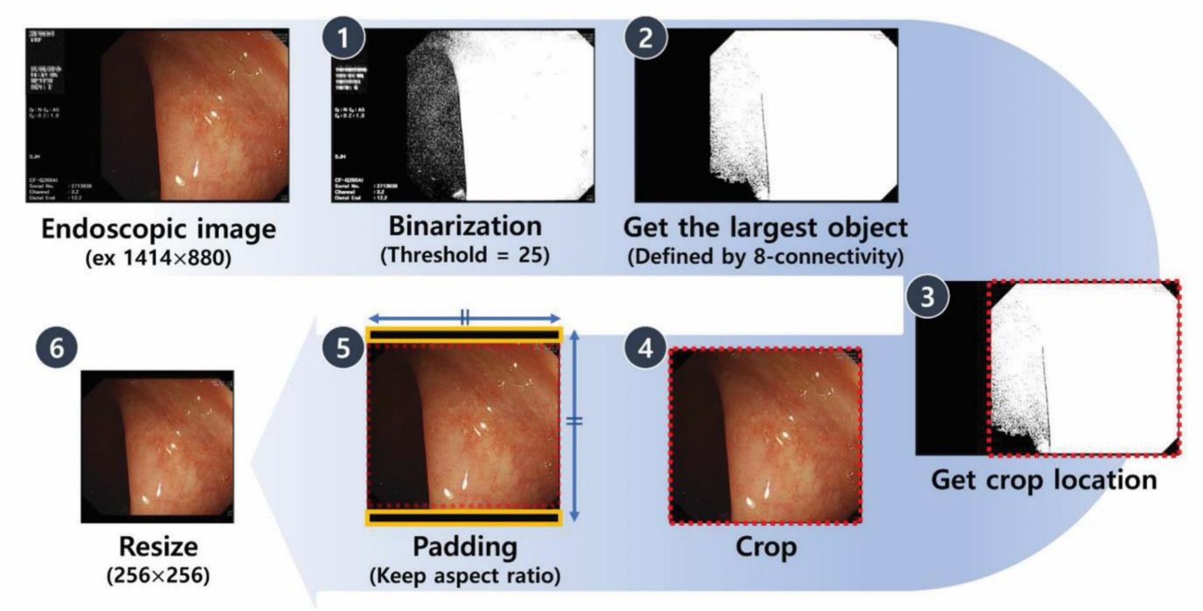

Supplementary Fig. 2. Length of endoscopic image.

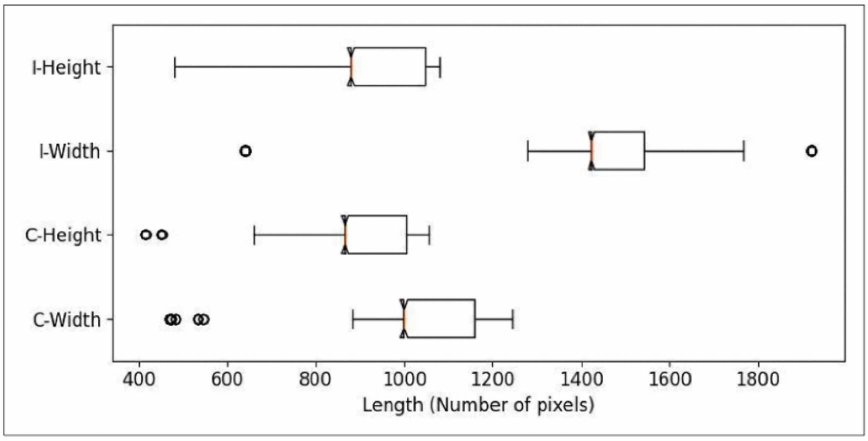

Supplementary Fig. 3. AUROC and AUPRC of our model in 12-fold cross validation.

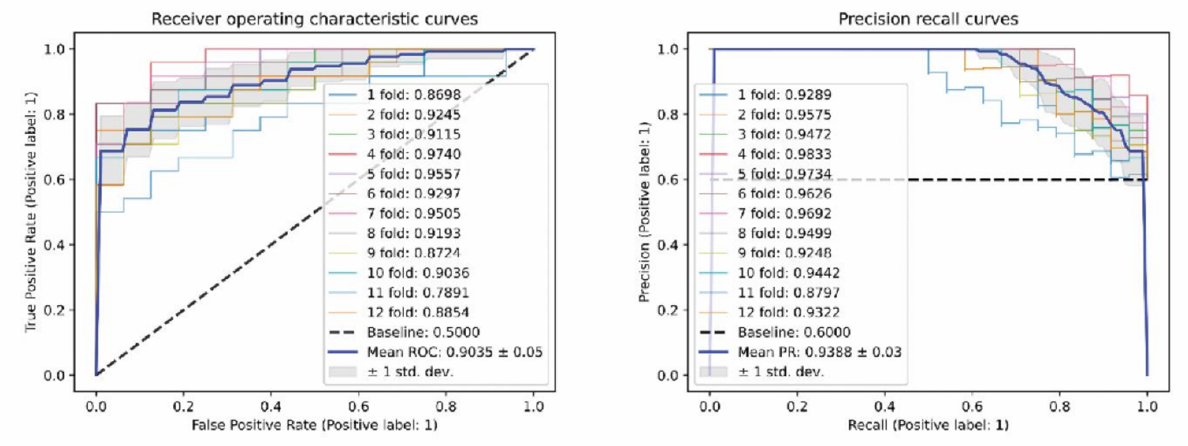

Supplementary Fig. 4. Confusion matrices of novice group and our AI model.

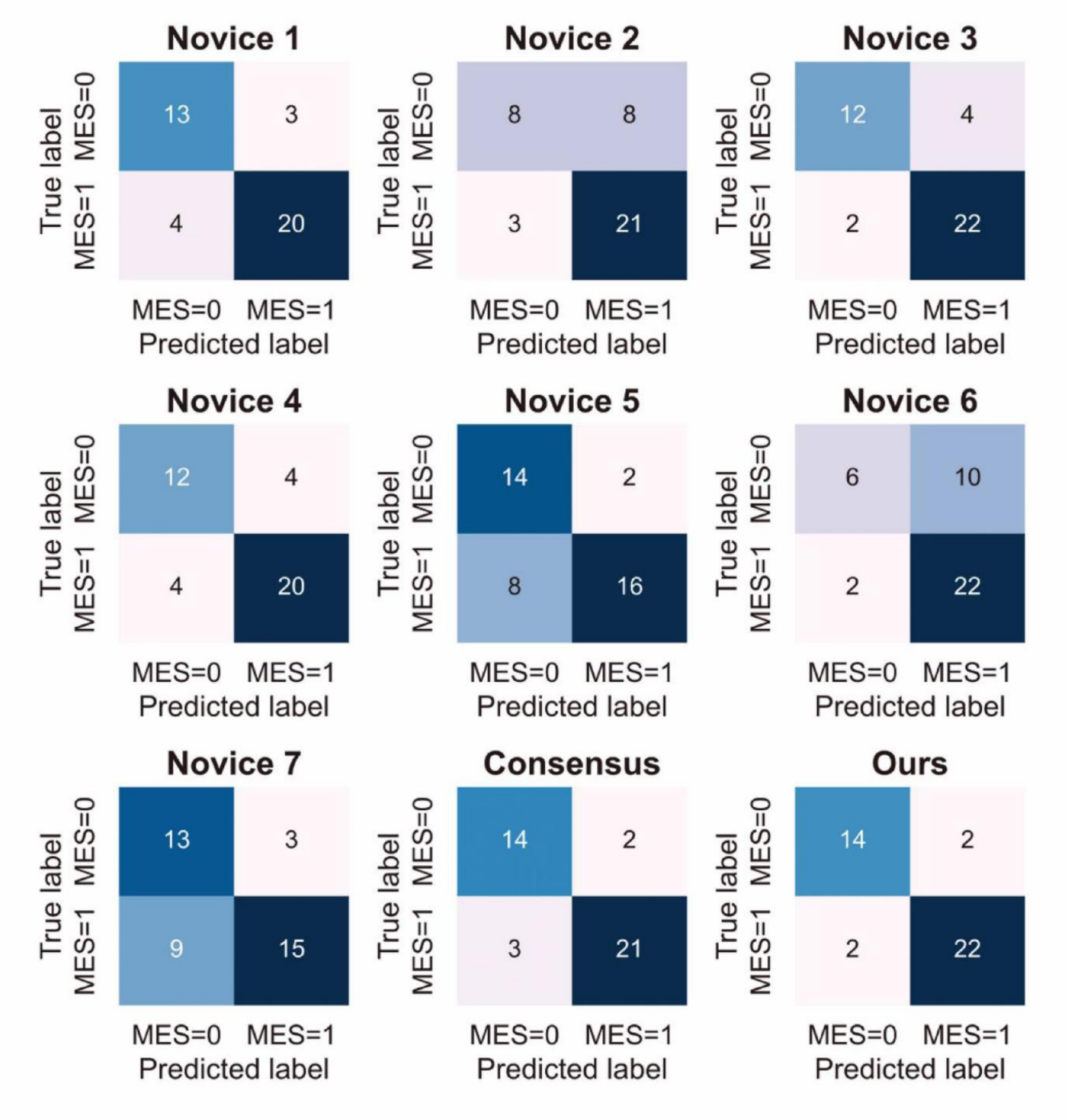

Supplementary Fig. 5. (A) Confusion Matrix on MES 0 vs MES 1 by external test (B) Confusion Matrix for other ulcerative colitis positive cases (MES 1~2, MES 2, MES 2~3, MES 3) in the external test set.

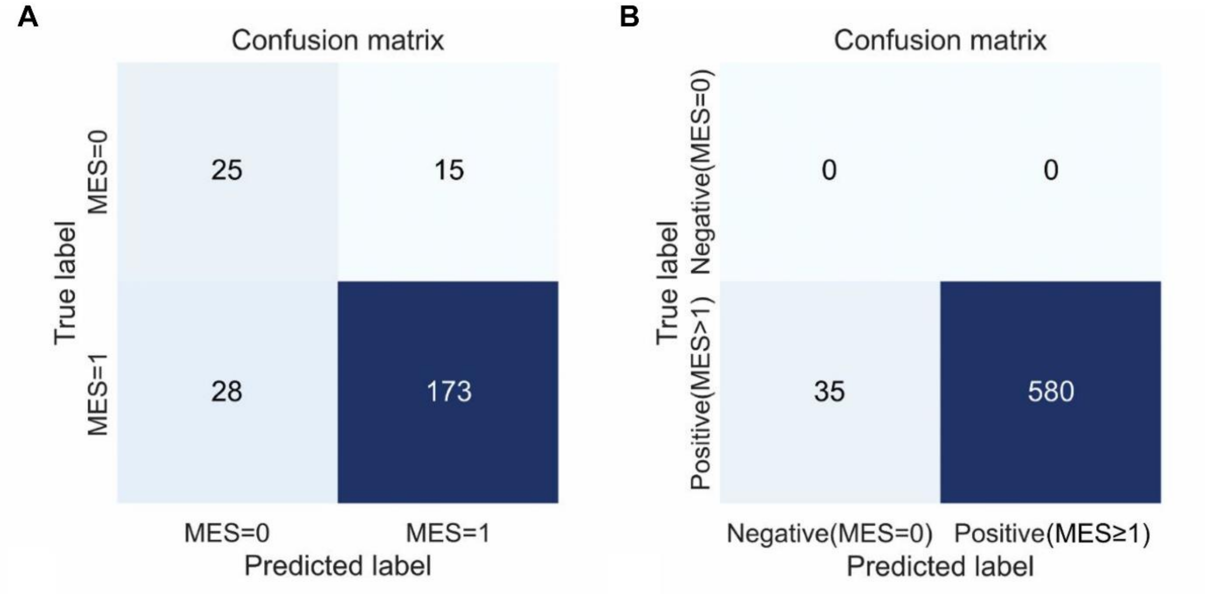

Supplement: Supplementary file 1 — Supplementary Information. [file 41598_2023_38206_MOESM1_ESM.pdf]
